# Supplementary material for: Glycolate combats massive oxidative stress by restoring redox potential in Caenorhabditis elegans
Source: Commun Biol. 2021 Feb 1;4:151. doi: 10.1038/s42003-021-01669-2 (PMC7851149; doi:10.1038/s42003-021-01669-2)
Supplement: Supplementary file 2 — Supplementary Information [file 42003_2021_1669_MOESM2_ESM.pdf]

Supplementary Information

Glycolate combats massive oxidative stress by restoring redox potential in *Caenorhabditis elegans*

Veronica Diez[1], Sofia Traikov[1], Kathrin Schmeisser[1], Akshay K. Das Adhikari[1] and Teymuras V. Kurzchalia[1*]

[1] Max Planck Institute of Molecular Cell Biology and Genetics, Dresden, Germany

* Correspondence to: kurzchalia@mpi-cbg.de

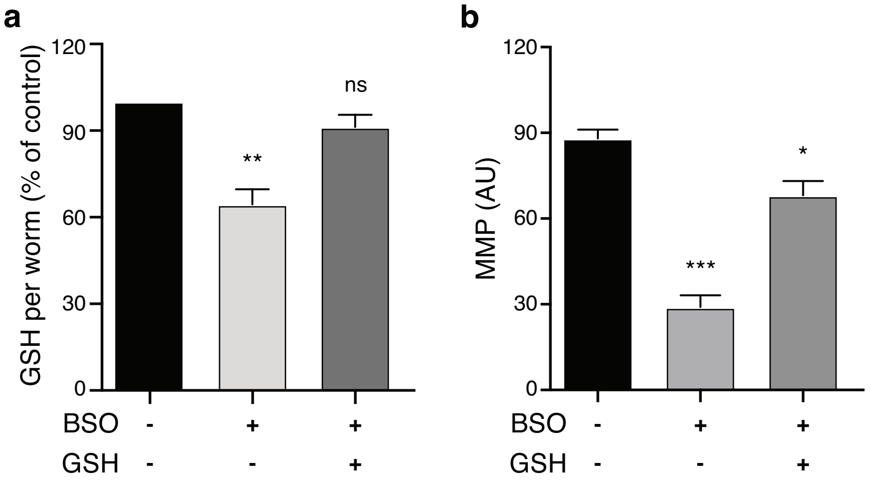

Supplementary Figure 1. BSO and GSH supplementation have opposite effects on relative amounts of GSH and MMP. Levels of GSH (a) and mitochondrial membrane potential (MMP) (b) were quantified after 2 days of incubation at 15°C from L1 stage with or without 0.5 mM BSO and 1mM GSH. Error bars represent standard error of the mean ($n = 3$).

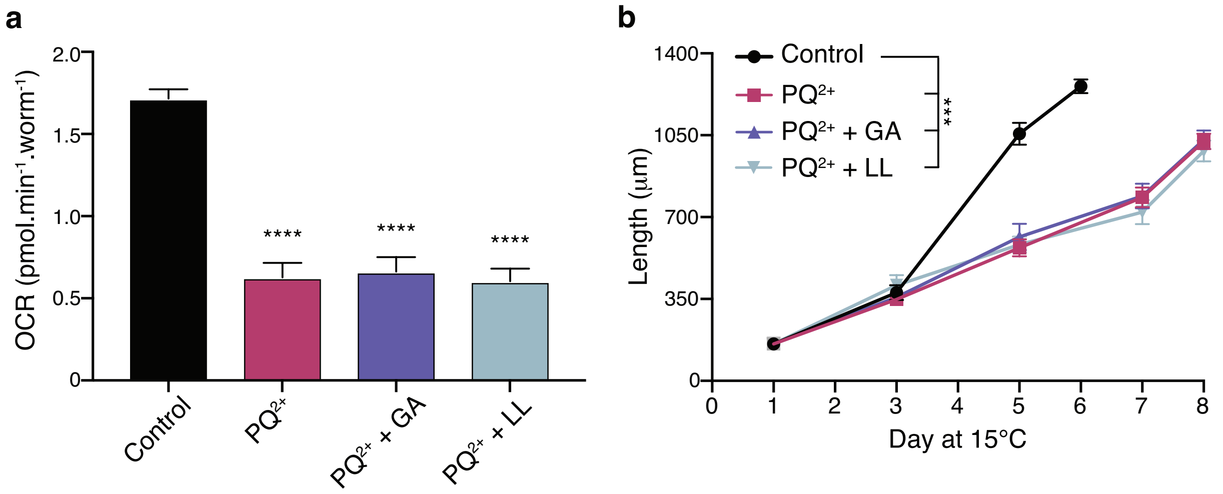

Supplementary Figure 2. Glycolate cannot rescue respiration and developmental rates of PQ^{2+}-exposed animals. a) Respiration rates in terms of oxygen consumption rate (OCR) determined after 2 days of incubation at 15°C from L1 stage with or without PQ^{2+}, GA and L-lactate. OCR was calculated using a Seahorse Analyzer and normalized to number of worms. b) Growth curves of worms incubated under the same conditions as in (a). The lengths of at least 20 animals per condition were determined every day and averaged for each experiment. Error bars represent standard error of the mean ($n = 5$). 200 μM PQ^{2+}, 10mM glycolate (GA), 10mM L-lactate (LL).

```
Score =  116 bits (290), Expect = 2e-26,   Method: Compositional matrix adjust.
Identities = 75/238 (31%), Positives = 120/238 (50%), Gaps = 9/238 (3%)

GRHPR_HUMAN  63  VDKRILDAAGANLKVISTMSVGIDHLALDEIKKRGIRVGYTPDVLTDTTAELAVSLLLTT 122
                 +   +L A+   LK++     G+D++ +       I V  TP   + + AEL  +L+L+
C31C9.2_CELE 58  ITAELLAASAGKLKLVGRAGTGVDNIDVPAASANKILVMNTPQANSRSAAELTCTLILSL 117

GRHPR_HUMAN  123 CRRLPEAIEEVKNGGWTSWKPLWLCGYGLTQSTVGIIGLGRIGQAIARRLKPFGVQRFLY 182
                  R +P+A   +K G W     +    YG    T+ ++GLGRIG  +A RL+ FG++
C31C9.2_CELE 118 SRHVPQAAASMKAGKWARKDFMGEEVYG---RTLAVLGLGRIGSEVAVRLQAFGMK---V 171

GRHPR_HUMAN  183 TGRQPR--PEEAAEFQAEFVSTPELAAQSDFIVVACSLTPATEGLCNKDFFQKMKETAVF 240
                  G  P    E+A     E +S  ++  Q+D+I V   L   TE L NK+   K K+
C31C9.2_CELE 172 IGFDPMVTKEQAEAKNIELLSLEQIWPQADYITVHVPLIKQTENLINKETLAKCKKGVRI 231

GRHPR_HUMAN  241 INISRGDVVNQDDLYQALASGKIAAAGLDVTSPEPLPTNHPLLTLKNCVILPHIGSAT 298
                 IN++RG +VN+ DL ++L +G    A  DV   EP PT   L+     +  PH+G++T
C31C9.2_CELE 232 INVARGGIVNEVDLVESLNAGHAKGAAFDVFEQEP-PTFRELIDHPLVIATPHLGAST 288
```

Supplementary Figure 3. Sequence alignment between the human Glyoxylate Reductase /Hydroxypyruvate reductase (GRHPR_HUMAN) and the protein sequence corresponding to C31C9.2 from *C. elegans* (C31C9.2_CELE). The alignment was carried out using the Blast tool from wormbase.org.

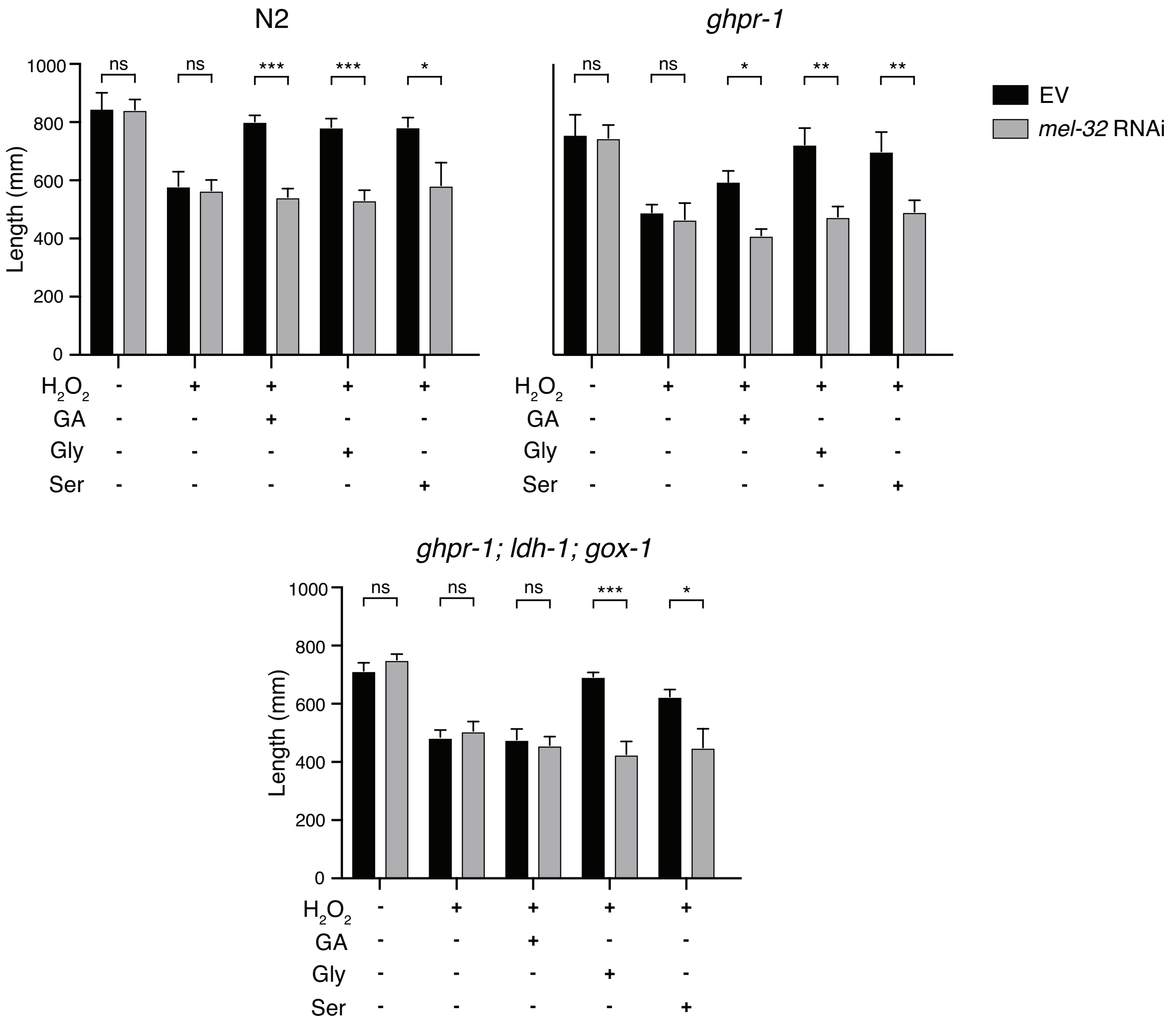

Supplementary Figure 4. Restoration of developmental rate by glycine and L-serine in mutants unable to metabolize glycolate to glyoxylate is lost upon inactivation of SHMT. Size of N2, *ghpr-1* and *ghpr-1, ldh-1, gox-1* worms fed with empty vector (EV) or RNAi bacteria against *mel-32*. Animals were incubated at 15°C from L1 stage for 4 days with or without 100 mM H_2O_2 together with 10 mM glycolate (GA), 100 μM glycine or 500 μM L-serine. The lengths of at least 20 animals per condition were determined and averaged for each experiment. Error bars represent standard error of the mean (*n* = 3).

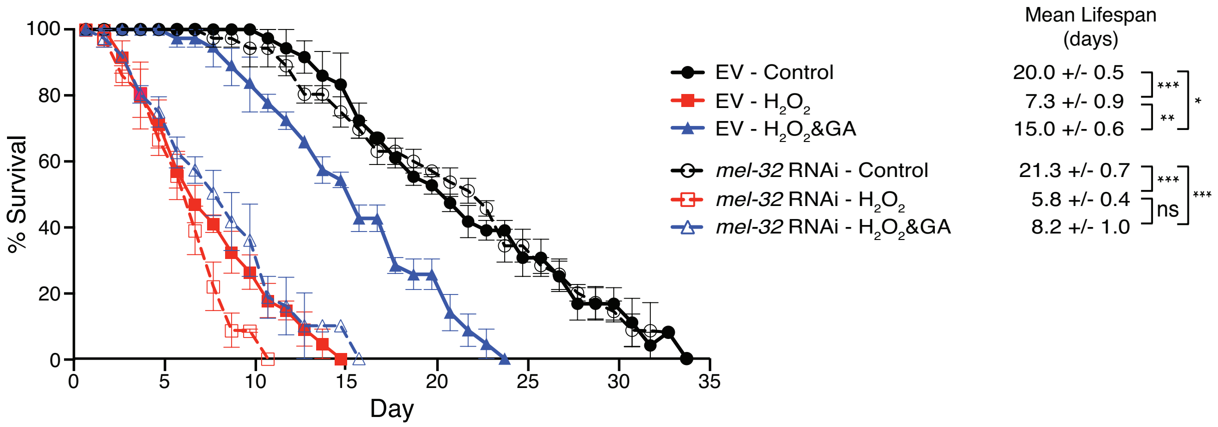

Supplementary Figure 5. Glycolate-mediated rescue of lifespan is lost in *mel-32* knockdown worms. Lifespan analysis without FUdR of N2 worms that had been exposed to H_2O_2 and 10mM GA, as indicated, during larval development and that were fed with empty vector (EV) or *mel-32* RNAi bacteria all along the growth curve. Error bars represent standard error of the mean (n = 3).

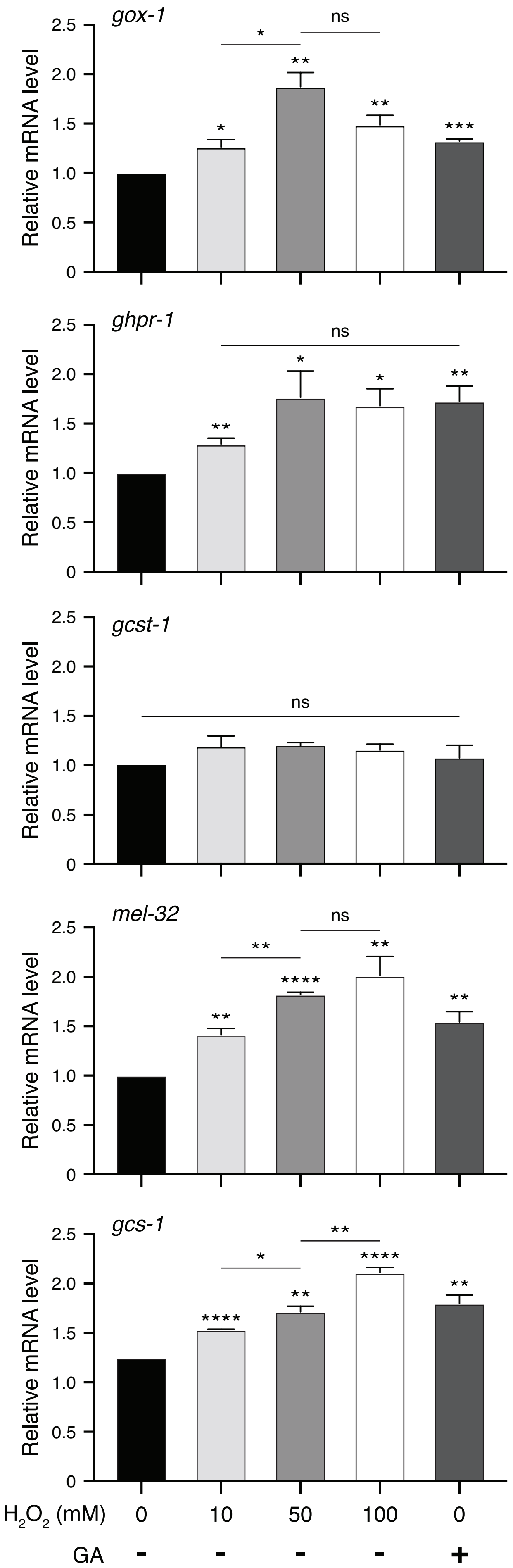
gox-1
ghpr-1
gcst-1
mel-32
gcs-1
Relative mRNA level
2.5
2.0
1.5
1.0
0.5
0
ns
*
**
***
****
H2O2 (mM)
0
10
50
100
0
GA
-
+

Supplementary Figure 6. H_2O_2 and glycolate induce the expression of glycolate metabolization-associated genes. Relative mRNA expression levels of *gox-1*, *ghpr-1*, *gcst-1*, *mel-32* and *gcs-1* were detected by reverse-transcription quantitative polymerase chain reaction. Animals were incubated at 15°C from L1 stage for 3 days with or without H_2O_2 or 10 mM glycolate (GA). Error bars represent standard error of the mean (n = 3). from worms incubated under different conditions as indicated from L1.

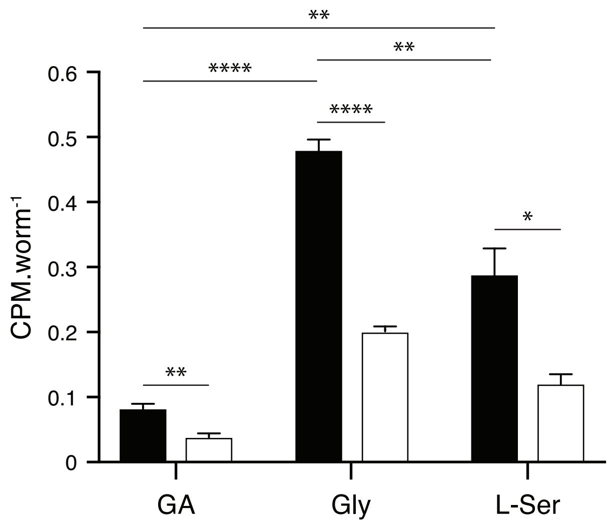

Supplementary Figure 7. Glycine and L-serine uptake by N2 worms are higher than glycolate. Animals were incubated at 15°C from L1 stage for 3 days with (white bars) or without 100 mM H_2O_2 (black bars) together with 10 µCi [1-^{14}C]-glycolate, [1-^{14}C]-glycine or L-[1-^{14}C]-serine Error bars represent standard error of the mean (n = 3).
